# The Tick Saliva Peptide HIDfsin2 TLR4-Dependently Inhibits the Tick-Borne Severe Fever with Thrombocytopenia Syndrome Virus in Mouse Macrophages

**DOI:** 10.3390/antibiotics13050449

**Published:** 2024-05-15

**Authors:** Luyao Wang, Yishuo Liu, Rui Pang, Yiyuan Guo, Yingying Ren, Yingliang Wu, Zhijian Cao

**Affiliations:** 1 National “111” Center for Cellular Regulation and Molecular Pharmaceutics, Key Laboratory of Fermentation Engineering (Ministry of Education), Hubei University of Technology, Wuhan 430068, China; 2018202040036@whu.edu.cn; 2 State Key Laboratory of Virology, College of Life Sciences, Wuhan University, Wuhan 430072, China; sixone@whu.edu.cn (Y.L.); 2018302050149@whu.edu.cn (R.P.); 2019202040010@whu.edu.cn (Y.G.); 2020202040046@whu.edu.cn (Y.R.); ylwu@whu.edu.cn (Y.W.); 3 Shenzhen Research Institute, Wuhan University, Shenzhen 518057, China

**Keywords:** tick saliva peptide, SFTSV, TLR4, tick–host–virus interactions, antiviral peptide

## Abstract

Ticks transmit a variety of pathogens to their hosts by feeding on blood. The interactions and struggle between tick pathogens and hosts have evolved bilaterally. The components of tick saliva can directly or indirectly trigger host biological responses in a manner that promotes pathogen transmission; however, host cells continuously develop strategies to combat pathogen infection and transmission. Moreover, it is still unknown how host cells develop their defense strategies against tick-borne viruses during tick sucking. Here, we found that the tick saliva peptide HIDfsin2 enhanced the antiviral innate immunity of mouse macrophages by activating the Toll-like receptor 4 (TLR4) signaling pathway, thereby restricting tick-borne severe fever with thrombocytopenia syndrome virus (SFTSV) replication. HIDfsin2 was identified to interact with lipopolysaccharide (LPS), a ligand of TLR4, and then depolymerize LPS micelles into smaller particles, effectively enhancing the activation of the nuclear factor kappa-B (NF-κB) and type I interferon (IFN-I) signaling pathways, which are downstream of TLR4. Expectedly, TLR4 knockout completely eliminated the promotion effect of HIDfsin2 on NF-κB and type I interferon activation. Moreover, HIDfsin2 enhanced SFTSV replication in TLR4-knockout mouse macrophages, which is consistent with our recent report that HIDfsin2 hijacked p38 mitogen-activated protein kinase (MAPK) to promote the replication of tick-borne SFTSV in A549 and Huh7 cells (human cell lines) with low expression of TLR4. Together, these results provide new insights into the innate immune mechanism of host cells following tick bites. Our study also shows a rare molecular event relating to the mutual antagonism between tick-borne SFTSV and host cells mediated by the tick saliva peptide HIDfsin2 at the tick–host–virus interface.

## 1. Introduction

After mosquitoes, ticks are the class of arthropods that carry the second largest number of pathogens [1]. The ability of ticks to transmit pathogens is partly due to the complex blood-sucking mechanism and evolved saliva components of ticks [2]. Complex immune interactions occur at the tick–host–pathogen interface, involving ticks’ immune escape and host immune defenses [3,4]. Among the problems caused by tick bites, infection with viruses is the main risk [5]. In severe cases, patients become delirious and suffer from multiple organ dysfunction, resulting in treatment being ineffective [5]. To sense and limit viral infection, mammalian cells defend themselves through innate and acquired immunity. Innate immunity acts as the first barrier, acting through the inflammatory response and the production of interferon (IFN-α/β) [6]. The early antiviral defense mechanism of the infected cells is based on the recognition of pathogen-associated molecular patterns (PAMPs). They trigger the activation of pattern-recognition receptors (PRRs), further transmit external signals and induce the production of transcription factors, thereby playing an antiviral role [6,7].

Toll-like receptors (TLRs) are a type of pattern-recognition receptor that sense a wide range of microbial ligands. TLR activation is characterized by the activation of inflammatory responses and the expression of other immune genes, involving the release of cytokines and the activation of interferon signaling pathways [8,9,10]. To date, 10 TLRs have been identified in humans, of which TLR4 is the most widely studied [11]. TLR4 activates the MAPK and NF-κB signaling pathways, inducing the production of pro-inflammatory cytokines by recruiting downstream myeloid differentiation factor 88 (MyD88) and can also activate downstream interferon signaling pathways by recruiting TIR-domain-containing adapter-inducing interferon-β (TRIF) [12].

TLR4 is involved in antiviral innate immunity in the recognition of viral infections and activation of downstream signaling pathways. In one study, pseudorabies virus (PRV) infection activated the TLR2/TLR3/TLR4/TLR5-NF-κB axis and upregulated the expression of the melanoma 2 (AIM2) inflammasome and gasdermin-d (GSDMD), further enhancing the inflammatory responses of mice, which play an important role in host defense against PRV infection [13]. The interaction between *Strongylocentrotus nudus* egg polysaccharide (SEP) and TLR4 induced the activation of the downstream molecules NF-κB and interferon regulatory factor 3 (IRF3), and further induced the production of the inflammatory cytokines and IFN-β, thereby inhibiting hepatitis B virus (HBV) replication in vivo and in vitro [14]. These findings suggest that TLR4 plays an important host-protective role in viral recognition.

SFTSV is a negative-strand RNA virus usually carried by the tick *Haemaphysalis longicornis*. Retinoic acid-inducible gene I (RIG-I)-like receptor and TLRs have been reported to be essential for SFTSV recognition, and the deletion of IFN-β promoter stimulator 1 (IPS-1) and MyD88 genes inhibits IFN-I production, increasing the viral loads in the serum [15]. Many peptides have been found to act as immunomodulators [16]. It has been reported that one peptide derived from mannose-binding lectin can inhibit LPS-induced TLR4/NF-κB activation, thereby suppressing the generation of inflammation [17]. Another viral inhibitory peptide of TLR4 (VIPER) can interact with MyD88 adaptor-like (Mal) and TRIF-related adaptor molecule (TRAM), two adaptor proteins of TLR4, blocking TLR4-mediated MAPK and transcription factor activation [18]. The peptide components secreted by tick salivary glands are easily understood as participating in the complex immune interactions that occur at the tick–host–virus interface. However, specific tick saliva peptides and the regulatory mechanisms involved in tick–host–virus interactions are unknown.

In this study, we found that the antiviral innate immunity of mouse macrophages was enhanced by the sensing of the tick saliva peptide HIDfsin2 through the TLR4 signaling pathway, thereby restricting the replication of the tick-borne SFTSV. HIDfsin2 was revealed to interact with LPS and depolymerize LPS aggregates. HIDfsin2 then activated the NF-κB signaling pathway and promoted the nuclear translocation of p65, upregulating the expression of inflammatory cytokines. Additionally, HIDfsin2 also upregulated IFN-β and subsequently upregulated related IFN-stimulated genes (ISGs). In contrast, TLR4 knockout completely eliminated the promoting effect of HIDfsin2 on inflammatory cytokines and IFN-β. Furthermore, we found that treatment with HIDfsin2 inhibited the replication of SFTSV in TLR4-expressing mouse peritoneal macrophages (MPMs), while HIDfsin2 promoted SFTSV replication in TLR4-knockout mouse macrophages, which is consistent with our previous finding that HIDfsin2 promoted the replication of tick-borne SFTSV in A549 and Huh7 cells with low expression of TLR4 by hijacking and activating p38 MAPK [19]. In brief, we found that the TLR4 signaling pathway of mouse macrophages senses the tick saliva peptide HIDfsin2, restricting tick-borne SFTSV infection.

## 2. Results

### 2.1. HIDfsin2 Inhibited SFTSV Replication in MPMs

**Figure 1:**
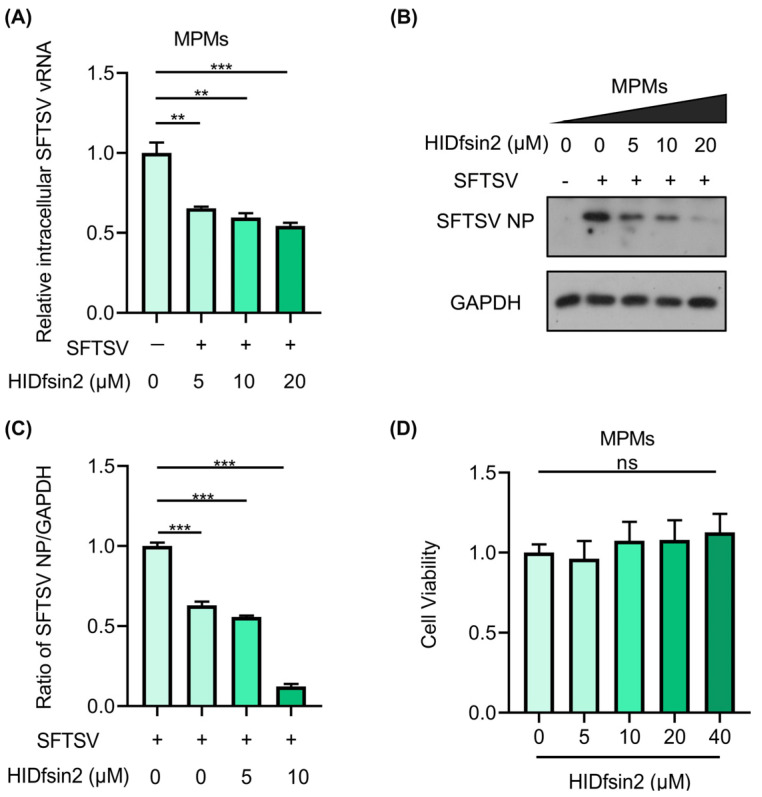
HIDfsin2 suppressed SFTSV replication in MPMs. (**A**,**B**) The inhibitory effect of HIDfsin2 on SFTSV replication in MPMs. MPMs were mixed with different concentrations of HIDfsin2 and pre-incubated for 1 h, and then MPMs were infected with SFTSV at an MOI = 1. After 72 h, cell samples were collected, and the SFTSV vRNA and NP protein levels were detected via qRT-PCR (**A**) and Western blotting (**B**), respectively. (**C**) The ratio of SFTSV NP to GAPDH was analyzed using ImageJ software. (**D**) The effect of HIDfsin2 on the toxicity of MPMs. Different concentrations of HIDfsin2 sample solutions were established; HIDfsin2-free solutions were used as negative controls and blank groups were set up without cells. After incubation for 48 h, the absorbance at 450 nm was measured. Data from three independent experiments were collected and analyzed, and are presented as the means ± SD. ns, no significance. ** *p* < 0.01. *** *p* < 0.001.

The interactions and struggle between tick pathogens and hosts have evolved bilaterally. From the perspective of host immunity, hosts develop antiviral strategies to restrict pathogens associated with tick bites. It is still unclear how HIDfsin2 affects the replication of SFTSV in host immune cells. To investigate this, the effect of HIDfsin2 on tick-borne SFTSV replication in MPMs was studied. The experimental results indicated that, with increasing HIDfsin2 concentrations, the expression of SFTSV NP at the both RNA (Figure 1A) and protein (Figure 1B) levels decreased significantly. Furthermore, the SFTSV NP/GAPDH ratio, as shown in Figure 1C, was analyzed using ImageJ v1.52 software, and the results showed that HIDfsin2 dose-dependently inhibited SFTSV replication (Figure 1C). The cell viability results proved that, when the concentration of HIDfsin2 was less than 20 μM, HIDfsin2 had no cytotoxicity in relation to MPMs (Figure 1D). These results indicate that the tick saliva peptide HIDfsin2 inhibits the replication of tick-borne SFTSV in MPMs, contrary to its promoting effect on SFTSV in A549/Huh7 cells.

### 2.2. HIDfsin2 Promoted SFTSV Replication in TLR4-Knockout MPMs

**Figure 2:**
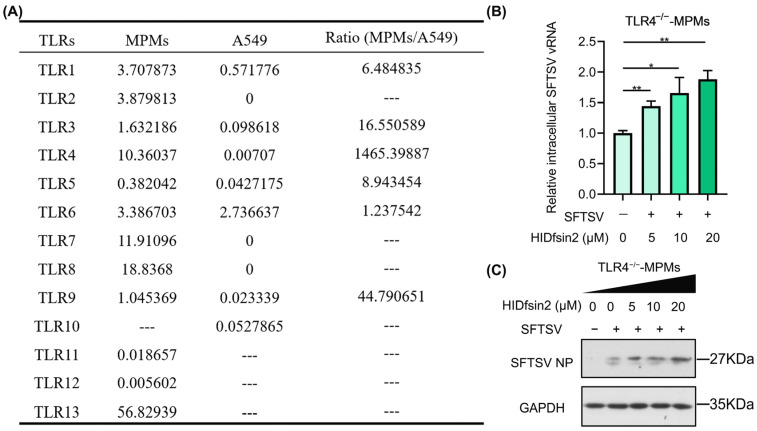
HIDfsin2 promoted SFTSV replication in TLR4^−/−^-MPMs. (**A**) Different expression profile of TLR4 in MPMs and A549 cells. RNA-Seq transcripts of MPMs (GSM3637970/GSM3637976) and A549 cells (GSM5610405/GSM5610406) were obtained from the GEO database, and their TLR gene expression profiles were annotated. (**B**,**C**) Dose-dependent promoting effect of HIDfsin2 on the replication of SFTSV in TLR4-knockout MPMs. TLR4^−/−^-MPMs were mixed with different concentrations of HIDfsin2 and pre-incubated at 37 °C for 1 h, and then infected TLR4^−/−^-MPMs cells were infected with SFTSV at an MOI = 1. After 72 h, cell RNA and protein samples were collected, and SFTSV vRNA (**B**) and SFTSV NP protein (**C**) were detected, respectively. * *p* < 0.05. ** *p* < 0.01.

To further explore the reason for the opposite effect of HIDfsin2 on SFTSV in MPMs and A549 cells, we analyzed the gene expression profiles of MPMs and A549 cells. We searched the RNA-Seq transcripts of MPMs (GSM3637970/GSM3637976) [20] and A549 cells (GSM5610405/GSM5610406) [21] from the GEO database and annotated their gene expression profiles. Macrophages are important immune cells that express a variety of innate immune receptors, such as TLRs, lectin receptors, and inflammasomes, which are located on the cellular membrane, in the endosomal compartment, and in the cytoplasm, respectively [22]. Based on the role of HIDfsin2 outside the cellular membrane and the importance of TLRs in innate immunity, we focused on comparing the expression of TLRs on the cell membrane of MPMs and A549 cells. The comparative analysis showed that TLR4 showed significantly different expression patterns in MPMs and A549 cells. TLR4 is highly expressed in MPMs, but its expression is extremely low in A549 cells. The fragments per kilobase million (FPKM) value of TLR4 in MPMs is 1465-fold higher than the FPKM value of TLR4 in A549 cells (Figure 2A). These results suggest that TLR4 is possibly related to the opposite effect of HIDfsin2 on SFTSV in MPMs and A549 cells; however, the role and mechanisms of TLR4 in the relationship between HIDfsin2 and SFTSV still need to be further experimentally verified.

To validate whether TLR4 is involved in SFTSV inhibition by HIDfsin2 in MPMs, we obtained TLR4-knockout C57BL/6J mice from Professor Zan Huang (College of Life Sciences, Wuhan University, China) and then prepared monolayer mouse peritoneal macrophages from the TLR4-knockout (TLR4^−/−^) mice. Next, we investigated the effect of HIDfsin2 on SFTSV replication in TLR4^−/−^-MPMs. The experimental results indicated that HIDfsin2 concentration-dependently promoted the replication of SFTSV at the both RNA (Figure 2B) and protein levels (Figure 2C) in TLR4-knockout MPMs, contrary to the inhibitory effects of HIDfsin2 on SFTSV in wild-type (TLR4^+/+^) MPMs. This result was completely consistent with our previously published conclusion that HIDfsin2 promotes tick-borne SFTSV in A549 and Huh7 cells with extremely low expression of TLR4 [19]. All of these data suggest that TLR4 in the cell membranes of MPMs is a key factor, playing an important role in SFTSV inhibition via HIDfsin2.

### 2.3. HIDfsin2 Interacted with the TLR4 Ligand LPS and Depolymerized LPS Micelles

**Figure 3:**
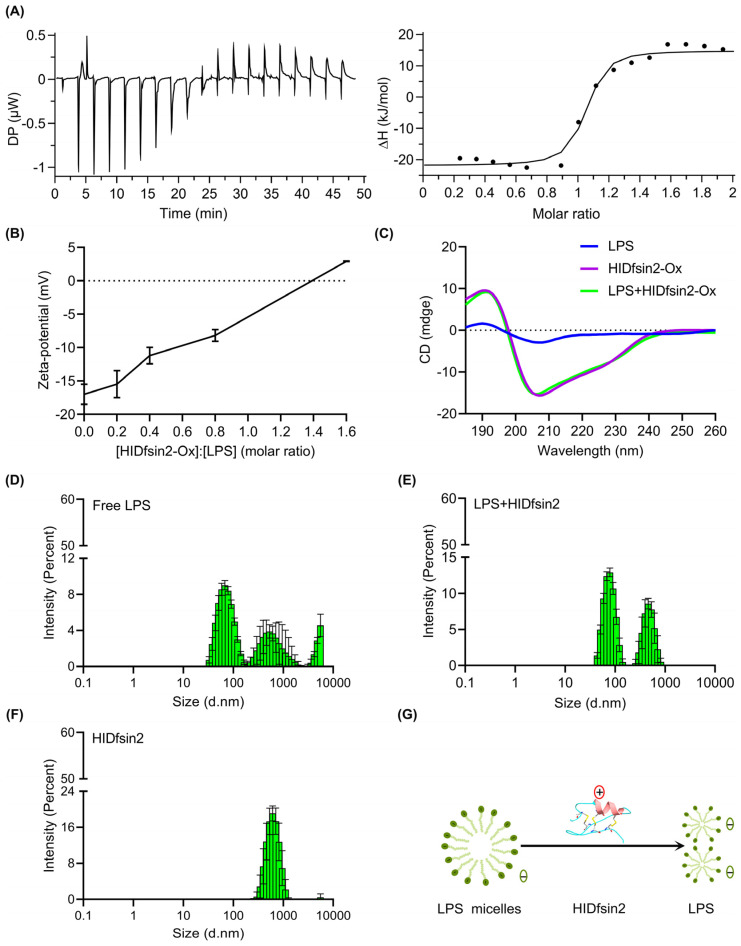
HIDfsin2 interacted with LPS and depolymerized LPS micelles. (**A**) Calorimetric titration profiles between HIDfsin2 and LPS. The left panel shows the peak of the titration, plotted as power against time. The panel on the right shows the combined heats of the corresponding interaction. Here, 200 μM LPS was titrated against HIDfsin2 at a concentration of 20 μM in double-distilled water (ddH_2_O). (**B**) The zeta potential between HIDfsin2 and LPS. HIDfsin2 and LPS were dissolved in ddH_2_O, and mixtures with different molar ratios were established and analyzed at room temperature. (**C**) The secondary structure of HIDfsin2 in LPS solution. Both HIDfsin2 and LPS were prepared with ddH_2_O at 25 μM and measured at room temperature. (**D**–**F**) The effect of the tick peptide HIDfsin2 on the size of LPS aggregates. The bar diagrams show the molecular particle sizes measured via DLS. LPS was prepared by aggregation into micelles and kept overnight at 4 °C with a final concentration of 140 μg/mL, and the particle size of free LPS was then measured (**D**). LPS was prepared by aggregation into micelles and kept overnight at 4 °C with a final concentration of 140 μg/mL, and 28 μM HIDfsin2 was added to the micelles. The mixture was placed at room temperature for 30 min, and then the molecular particle size of LPS was detected (**E**). HIDfsin2 was dissolved in phosphate-buffered saline (PBS), and its molecular particle size was measured (**F**). The standard deviation calculated from 3 runs is shown by error bars. (**G**) Schematic of LPS micelles’ depolymerization by HIDfsin2. The “+” in the red circle represents a positive charge, and the “−” symbols in the green circles represent negative charges.

TLR4 mediates the inhibition of the tick-borne SFTSV by the tick saliva peptide HIDfsin2 in MPMs. LPS is well known to be a specific ligand of TLR4. Moreover, there are many reports that cationic antimicrobial peptides can interact with LPS to exert antibacterial activities [23,24,25]. All of these clues led us to speculate that the tick saliva peptide HIDfsin2 may interact with LPS. To explore this issue, we detected the interaction between HIDfsin2 and LPS through isothermal titration calorimetry (ITC). The ITC curve showed that HIDfsin2 and LPS had obvious thermal changes (Figure 3A), and the thermodynamic parameters of the combination of HIDfsin2 with LPS were as follows: ΔH (kJ/mol) = −36.7 ± 1.79; −TΔS (kJ/mol) = −3.88; ΔG (kJ/mol) = −40.5; KD (M) = 80.0 × 10^−9^ ± 40.6 × 10^−9^. Combining these findings with the titration curve and thermodynamic parameters (ΔH < 0, ΔS > 0) clearly shows that the bonding reaction was slightly exothermic and driven primarily by a favorable change in entropy. The main driving force was electrostatic action. “ΔG < 0” indicated that the LPS–HIDfsin2 interaction was spontaneous. Furthermore, the degree of binding between HIDfsin2 and LPS was measured in terms of the zeta potential (Figure 3B). The zeta potential of LPS was −17.00 mV. The potential value of the mixed system was close to 0 mV when the molar ratio of HIDfsin2 to LPS was about 1.4, while HIDfsin2 completely neutralized the negative charge of LPS when the molar ratio of HIDfsin2 to LPS was increased to 1.6. This resulted in the charge overcompensation phenomenon, which was completely consistent with the ITC results. To further investigate whether LPS affected the secondary structure of HIDfsin2, we analyzed the secondary structure using the CD method (Figure 3C). In an aqueous buffer, LPS presented a disordered structure (blue line), and the curve trend of HIDfsin2 in the aqueous buffer (purple line) coincided with that in LPS buffer (green line), indicating that LPS did not affect the secondary structure of HIDfsin2.

LPS is composed of lipids and polysaccharides. Lipids are hydrophobic, but polysaccharides are hydrophilic. Therefore, LPSs have amphiphilic characteristics, meaning that they usually form micelles in the aqueous phase. However, the formation of LPS micelles is thought to reduce LPS’s activation of TLR4. To further investigate HIDfsin2 interference with the structure of LPS micelles, we explored the effect of HIDfsin2 on the size of LPS aggregates using dynamic light scattering (DLS). As a control, the molecular particle size of HIDfsin2 was also measured. LPSs became macromolecular aggregates after being left overnight. The size of the LPS micelles ranged from 100 to 10,000 nm (Figure 3D). After HIDfsin2 was added, the molecular particle size of the LPS micelles decreased significantly (Figure 3E). HIDfsin2 also has a certain particle size (Figure 3F), which means that HIDfsin2 forms macromolecular aggregates in solution. This could be explained by the fact that HIDfsin2 is also an amphiphilic peptide. In conclusion, HIDfsin2 can depolymerize LPS micelles (Figure 3G).

### 2.4. HIDfsin2 Promoted NF-κB Activation and Inflammatory Cytokine Expression

**Figure 4:**
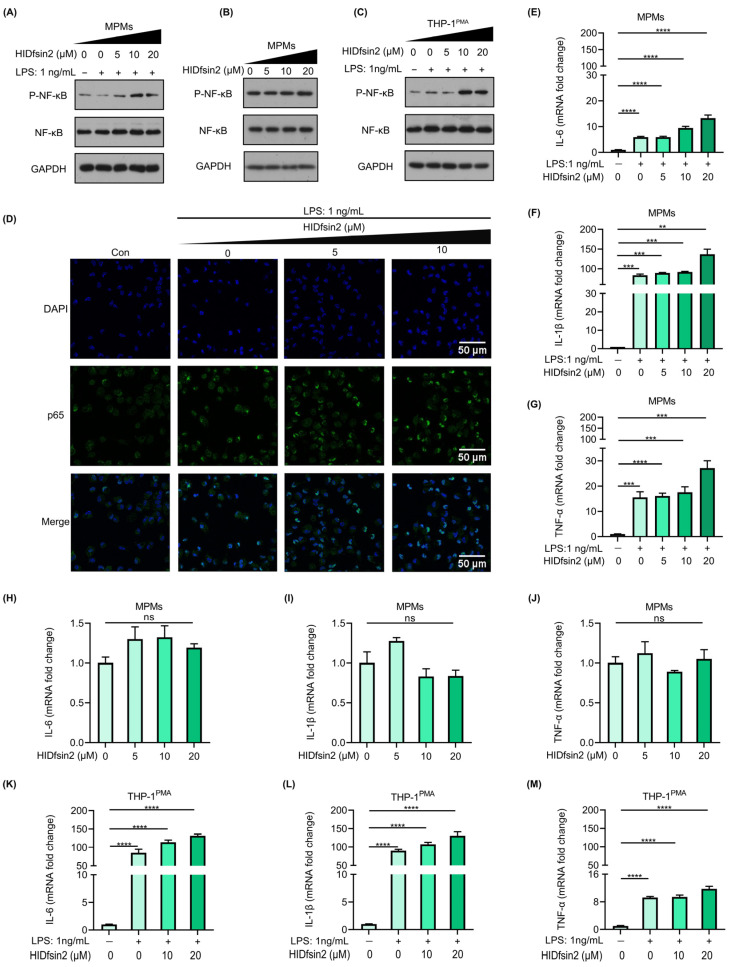
HIDfsin2 promoted NF-κB activation and inflammatory cytokine expression. (**A**–**C**) The promoting effect of HIDfsin2 on NF-κB activation in LPS-stimulated macrophages. MPMs (**A**,**B**) or THP-1^PMA^ (**C**) cells were incubated overnight with serum-free RPMI 1640 medium. Different concentrations of HIDfsin2 and LPS were incubated at 37 °C for 2 h and then added into cells, or HIDfsin2 alone was used to treat MPMs (**B**). After 24 h, the intracellular protein levels were detected by Western blotting. (**D**) The promoting effect of HIDfsin2 on p65 nuclear translocation in MPMs. MPMs were pre-treated with serum-free RPMI 1640 medium for 12 h. HIDfsin2 and LPS were incubated at 37 °C for 2 h and then added to MPMs. After 30 min, the cells were fixed in 4% paraformaldehyde, and immunofluorescence staining was performed using an anti-p65 antibody (green). The nucleus was stained with DAPI (blue). (**E**–**G**) The promoting effect of HIDfsin2 on the expression of inflammatory cytokines in LPS-stimulated MPMs. MPMs were pre-treated with serum-free medium for 12 h, and then LPS and HIDfsin2 were added simultaneously. After 8 h, RNA was collected and the transcription levels of IL-6 (**E**), IL-1β (**F**), and TNF-α (**G**) were detected by qRT-PCR, respectively. (**H**–**J**) The effect of HIDfsin2 alone on the expression of inflammatory cytokines in MPMs. MPMs were pre-treated with serum-free medium for 12 h, and then HIDfsin2 was added. After 8 h, RNA was collected and the transcription levels of IL-6 (**H**), IL-1β (**I**), and TNF-α (**J**) were detected by qRT-PCR. (**K**–**M**) The promoting effect of HIDfsin2 on the expression of inflammatory cytokines in LPS-stimulated THP-1^PMA^ cells. THP-1^PMA^ cells were treated in the same way as MPMs, and the RNA levels of IL-6 (**K**), IL-1β (**L**), and TNF-α (**M**) were detected. Data represent the mean ± SD of at least three independent experiments. ns, no significance. ** *p* < 0.01. *** *p* < 0.001. **** *p* < 0.0001.

It was reported that the host defense peptide cathelicidin LL-37 binds to LPS and then promotes the response of epithelial cells to LPS by delivering LPS to TLR4-containing intracellular compartments [26]. In order to test whether the interaction between HIDfsin2 and LPS can activate the TLR4 signaling pathway, we detected the activation of NF-κB, which is downstream of TLR4. The results showed that HIDfsin2 enhanced the phosphorylation of NF-κB under the stimulation of 1 ng/mL LPS in MPMs (Figure 4A), but HIDfsin2 did not affect the expression of the total NF-κB protein (Figure 4B). Similarly, the phosphorylation of NF-κB was also increased by HIDfsin2 in THP-1^PMA^ cells (Figure 4C). Moreover, HIDfsin2 is not cytotoxic to THP-1^PMA^ cells at a concentration of 20 μM. To further verify the activation of NF-κB by HIDfsin2, the nuclear translocation of p65 was detected using a confocal laser scanning microscope. The experimental results indicated that HIDfsin2 dose-dependently promoted the nuclear translocation of p65 in MPMs (Figure 4D). These results indicate that the interaction between HIDfsin2 and LPS activates the NF-κB signaling pathway, which is downstream of TLR4.

Since HIDfsin2 interaction with LPS enhanced the phosphorylation of NF-κB, we further investigated whether HIDfsin2 affected the expression of the cytokines IL-6, IL-1β, and TNF-α, which are downstream of NF-κB. The results showed that HIDfsin2 could dose-dependently promote the expression of IL-6 (Figure 4E), IL-1β (Figure 4F), and TNF-α (Figure 4G) at the RNA level in 1 ng/mL LPS-stimulated MPMs. Moreover, HIDfsin2 alone did not induce the expression of these three cytokines (Figure 4H–J). Similarly, HIDfsin2 also significantly increased the expression of the three cytokines IL-6, IL-1β, and TNF-α in THP-1^PMA^ cells (Figure 4K–M). All of these results suggest that the interaction between HIDfsin2 and LPS can upregulate the expression of cytokines downstream of NF-κB.

### 2.5. HIDfsin2 Enhanced the Type I Interferon Pathway

**Figure 5:**
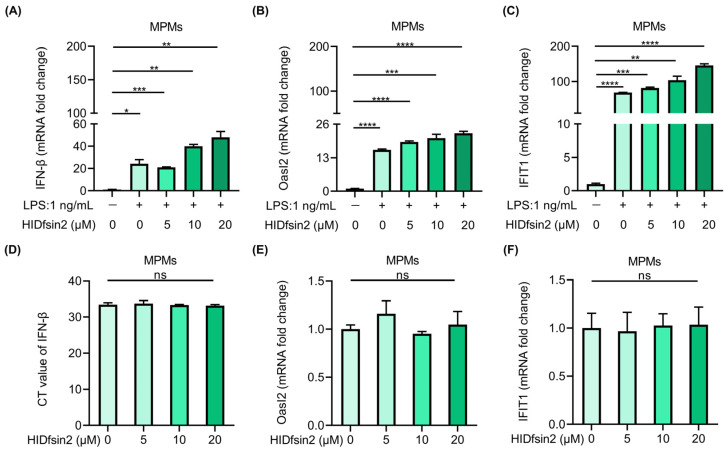
HIDfsin2 enhanced interferon and interferon-stimulated gene expression. (**A**–**C**). HIDfsin2 enhancement of IFN-β and interferon’s stimulation of genes expression in LPS-induced MPMs. MPMs were pre-treated with serum-free medium for 12 h, and then LPS and HIDfsin2 were added simultaneously. After 8 h, the transcription levels of IFN-β (**A**), Oasl2 (**B**), and IFIT1 (**C**) were detected by qRT-PCR. (**D**–**F**) The effect of HIDfsin2 alone on type I interferon pathway in MPMs. After the same treatment, different concentrations of HIDfsin2 were added to MPMs. After 8 h, the expression of IFN-β (**D**), Oasl2 (**E**), and IFIT1 (**F**) was detected by qRT-PCR. ns, no significance. * *p* < 0.05. ** *p* < 0.01. *** *p* < 0.001. **** *p* < 0.0001.

The results shown above indicated that HIDfsin2 interacted with LPS to activate signaling pathways downstream of TLR4. Firstly, NF-κB activation triggered an important downstream signaling pathway that led to the significant upregulation of inflammatory factors. Secondly, TLR4’s interaction with its ligand may also trigger the activation of the type I interferon signaling pathway [27]. Therefore, we detected the effect of HIDfsin2 on the interferon signaling pathway during stimulation with LPS. The subsequent results showed that HIDfsin2 could obviously upregulate the expression of IFN-β (Figure 5A) and promote the expression of two interferon-stimulating genes (ISGs), Oasl2 (Figure 5B) and IFIT1 (Figure 5C), in a dose-dependent manner. However, when HIDfsin2 was added to cells alone, intracellular IFN-β expression was extremely low and did not change significantly (Figure 5D). Similarly, the expression of Oasl2 (Figure 5E) and IFIT1 (Figure 5F) was not regulated by HIDfsin2.

### 2.6. TLR4 Mediated the Enhancement of HIDfsin2 on NF-κB and Type I Interferon Activation

**Figure 6:**
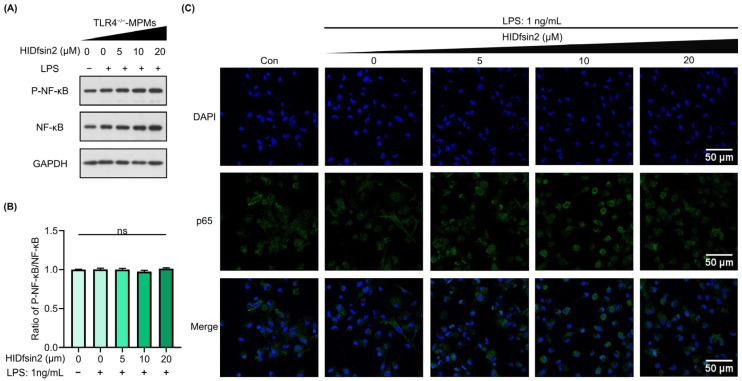
HIDfsin2 enhanced the TLR4-mediated activation of the NF-κB pathway. (**A**) HIDfsin2 depends on TLR4 to enhance the activation of the NF-κB pathway in MPMs. TLR4^−/−^-MPMs were pre-treated with serum-free medium for 12 h, and then LPS and HIDfsin2 were added simultaneously. After 24 h, the expression of P-NF-κB p65 was detected by Western blotting. (**B**) The ratio of P-NF-κB to NF-κB was analyzed using ImageJ software. (**C**) HIDfsin2 depends on TLR4 to enhance the nuclear translocation of p65 in MPMs. TLR4^−/−^-MPMs were pre-treated with serum-free RPMI 1640 medium for 12 h; HIDfsin2 and LPS were incubated at 37 °C for 2 h and then added to TLR4^−/−^-MPMs. After 30 min, cells were fixed with 4% paraformaldehyde, and immunofluorescence staining was performed using an anti-p65 antibody (green). The cellular nucleus was stained with DAPI (blue). ns, no significance.

To further verify the role of TLR4 in HIDfsin2 ability to upregulate the NF-κB signaling pathway, we isolated peritoneal macrophages from TLR4-knockout C57BL/6J mice (TLR4^−/−^-MPMs). Additionally, the effect of HIDfsin2 on the phosphorylation of NF-κB and the nuclear translocation of p65 were investigated in relation to TLR4^−/−^-MPMs. The experimental results showed that LPS could not induce NF-κB phosphorylation after deletion of the TLR4 gene in MPMs (Figure 6A), and there was no visible difference in the P-NF-κB/NF-κB ratio with an increase in HIDfsin2 concentration (Figure 6B). Consistently, the nuclear translocation of p65 was not observed in TLR4^−/−^-MPMs (Figure 6C). The average fluorescence intensity of p65 did not change significantly with an increase in HIDfsin2 concentration. These data suggest that HIDfsin2 ability to enhance the activation of the NF-κB signaling pathway is dependent on the cell receptor TLR4.

**Figure 7:**
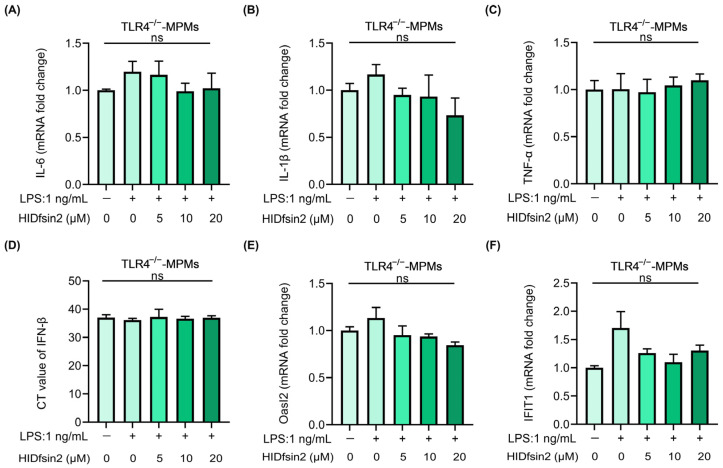
HIDfsin2 TLR4-dependently promoted the expression of inflammatory cytokines, IFN-β, and ISGs. (**A**–**C**) The effect of HIDfsin2 on the expression of inflammatory cytokines in TLR4^−/−^-MPMs. TLR4^−/−^-MPMs were pre-treated with serum-free medium for 12 h, and then LPS and HIDfsin2 were added simultaneously. After 8 h, the transcription levels of IL-6 (**A**), IL-1β (**B**), and TNF-α (**C**) were detected using qRT-PCR. (**D**–**F**) HIDfsin2 ability to enhance the expression of IFN-β and ISGs in MPMs depended on TLR4. After the same treatment, different concentrations of HIDfsin2 were added to TLR4^−/−^-MPMs. After 8 h, the expressions of IFN-β (**D**), Oasl2 (**E**), and IFIT1 (**F**) were detected using qRT-PCR. ns, no significance.

We further determined the role of TLR4 in the expression of inflammatory cytokines downstream of NF-κB and interferon and ISG genes, which was promoted by HIDfsin2. The experimental results showed that as the HIDfsin2 concentration was increased from 0 μM, 5 μM, and 10 μM to 20 μM in TLR4^−/−^-MPMs, significant changes in the expression of IL-6 (Figure 7A), IL-1β (Figure 7B), and TNF-α (Figure 7C) were not observed, in stark contrast to the results obtained for MPMs expressing TLR4. Similarly, when the TLR4 gene was knocked out in MPMs, IFN-β (Figure 7D), Oasl2 (Figure 7E), and IFIT1 (Figure 7F) were all not regulated by HIDfsin2. Altogether, these results suggest that TLR4 plays a key regulatory role in HIDfsin2 enhancement of the activation of both NF-κB and type I interferon pathways.

## 3. Discussion

Ticks are a class of obligate blood-sucking arthropods that carry a variety of pathogens [28]. Ticks’ salivary glands are key to tick-borne pathogens. In order to sustain blood feeding and pathogen transmission, tick-carried pathogens have evolved to resist and evade complex physiological host immunity and homeostasis responses. In addition, hosts respond to tick-borne pathogens or the components of tick saliva and further activate innate and adaptive immunity to combat these pathogens [29]. However, how hosts develop their defense strategies against tick-borne viruses during tick sucking is still unclear. Therefore, an in-depth understanding of the biological events involved in host defense against tick bites is of great significance for the prevention and treatment of tick-borne diseases.

In our study, we found that the tick salivary peptide HIDfsin2 inhibited tick-borne SFTSV replication in mouse macrophages. However, our previous study reported that HIDfsin2 promoted the replication of SFTSV in A549 and Huh7 cells [19]. Clearly, the same peptide has opposite biological effects in different cells. As important innate immune cells, macrophages play an important protective role in host innate immunity. Macrophages express a variety of innate immune receptors, such as TLRs, lectin receptors, and inflammasomes [22]. According to the characteristics of HIDfsin2 acting outside the cell membrane, we were inspired by the differences in the gene expression profiles of MPMs and A549 cells [20,21], and targeted the innate immune receptor TLR4 on the cell membrane. In contrast to the suppression of SFTSV replication in TLR4-expressing macrophages, HIDfsin2 promoted SFTSV in TLR4-knockout MPMs. This was completely consistent with the promoting effect observed in A549 and Huh7 cells, with extremely low expression of TLR4. In mouse macrophages, TLR4 enhanced the antiviral innate immunity in response to the tick saliva peptide HIDfsin2, thereby restricting the infection and transmission of the tick-borne SFTSV.

It was reported that many antimicrobial peptides have high affinity with LPS [30,31,32]. In our study, the ITC experiment indicated that HIDfsin2 can bind to LPS. The binding between HIDfsin2 and LPS further depolymerized LPS micelles into smaller particles. The activation of the downstream NF-κB signaling pathway and nuclear translocation of p65 were mediated by TLR4. The expression of inflammatory cytokines and IFN-β was upregulated. Accordingly, the above promoting effect disappeared in TLR4-knockout mouse macrophages. These results suggest that the macrophage TLR4 signaling pathway can recognize the tick saliva peptide component, further activating host innate immune responses and then inhibiting tick-borne pathogens.

We used a molecular model to visually elucidate the mechanism by which TLR4-mediated HIDfsin2 inhibits SFTSV replication in mouse macrophages (Graphical Abstract A) and the opposite effect of HIDfsin2 on SFTSV replication in infected cells with or without TLR4 (Graphical Abstract B). The opposite biological effects of HIDfsin2 on SFTSV replication in MPMs and A549 cells found by our group precisely reflect the relationship between the tick saliva peptide HIDfsin2 and tick-borne SFTSV from two perspectives: tick-borne viruses and hosts. The interactions and struggle between tick-borne viruses and hosts have evolved bilaterally. Tick-borne viruses try their best when attempting immune escape to promote infection and transmission. On the contrary, hosts continuously develop antiviral strategies to fight the infection and transmission of tick-borne viruses. At the tick–host–virus interface, tick-borne SFTSV and the mouse macrophages simultaneously utilize the same tick saliva peptide component HIDfsin2 to produce completely contrary bioactivities via different molecular mechanisms. Our study shows a rare molecular event concerning the mutual antagonism between this tick-borne virus and hosts.

However, this study has some limitations. Only one tick-borne virus, SFTSV, was explored in this study, and whether the molecular function of the tick saliva peptide HIDfsin2 applies to other tick-borne viruses remains to be determined. In addition, in the early stages of tick bites, salivary gland secretions disrupt the host’s immune response. At the same time, the compounds secreted in tick saliva also activate the host’s innate immune response. However, it takes time for hosts to recognize tick saliva compounds. Ultimately, the mechanism of bidirectional competition occurs at the tick–host interface. The threshold for HIDfsin2 to promote SFTSV or inhibit SFTSV replication at the tick bite interface is still unclear and requires further study.

## 4. Materials and Methods

### 4.1. Animals

C57BL/6J mice (SPF degree, 6–8 weeks) were purchased from the Hubei Provincial Center for Disease Control and Prevention of China. TLR4^−/−^-C57BL/6J mice (SPF degree, 16 weeks) were kindly provided by Professor Zan Huang (College of Life Sciences, Wuhan University, China). After the propagation of the 16-week-old TLR4^−/−^-C57BL/6J mice, MPMs were extracted from the offspring of TLR4^−/−^-C57BL/6J mice, which were 6–8 weeks old. All of the mice were kept in animal rooms with a light–dark cycle at 24 °C for 12 h. All of the experiments were aligned with the policies and recommendations of the Animal Welfare Ethics and Use Committee of the College of Life Sciences, Wuhan University; the approval code is WDSKY0201707-2. The date was 11 December 2021.

### 4.2. Cells and Viruses

Mouse peritoneal macrophages (MPMs) and human monocyte leukemia cells (THP-1 cells) (TIB-202) were maintained in RPMI 1640 medium (Thermo Fisher, Waltham, MA, USA, item no. 11875168) containing 10% fetal bovine serum (FBS) (Thermo Fisher, Waltham, MA, USA, item no. A5669801) and 1% penicillin/streptomycin (Merck, Rahway, NJ, USA, item no. TMS-AB2) at 37 °C with 5% CO_2_. Prior to viral infection, the THP-1 cells needed to be induced to differentiate using Phorbol 12-myristate 13-acetate (PMA) (MedChemExpress, Newark, NJ, USA, item no. HY-18739). The SFTSV strain was gifted by Professor Xuejie Yu from the School of Health Sciences, Wuhan University, China.

### 4.3. Reagents and Antibodies

The reduced form of HIDfsin2 was chemically synthesized by GL Biochem Co., Ltd. (Shanghai, China). Phosphatase inhibitors (TargetMol, Boston, MA, USA, item no. C0004) and protease inhibitors (TargetMol, Boston, MA, USA, item no. C0001) were purchased from TargetMol. LPS (Shanghai, China, item no. S1732-5mg) was acquired by Beyotime. Cell counting kit-8 (CCK-8) (Shanghai, China, item no. 40203ES60) was obtained from YeaSen. P-NF-κB p65 (Ser536) (93H1) rabbit mAb (the WB dilution ratio was 1:1000) (Cell Signaling Technology, Danvers, MA, USA, item no. #3033) and NF-κB p65 antibody (the WB dilution ratio is 1:1000) (Cell Signaling Technology, Danvers, MA, USA, item no. #8242) were Cell Signaling Technology (CST) products. The GAPDH monoclonal antibody (Proteintech, Rosemont, IL, USA, item no. 60004-1-lg) was ordered from Proteintech. The SFTSV NP rabbit polyclonal antibody was customized by AtaGenix Laboratories Co., Ltd. (Wuhan, China).

### 4.4. MPM Extraction

Mice aged 6–8 weeks were intraperitoneally injected with 1 mL of 4% thioglycolate medium every day for 3 consecutive days. The mice were euthanized via CO_2_ inhalation and then soaked in 75% ethanol for 1–2 min. Subsequently, the abdomens of these mice were cut open to expose the peritoneum, and 5 mL of pre-cooled PBS was injected into the peritoneum. The abdomens of these mice were gently touched for 2–5 min to collect intraperitoneal cells. After centrifugation at 1000 rpm for 6 min, the cells were re-suspended using RPMI 1640 medium. After 2–4 h, the non-adherent cells were washed away, and the adherent cells were monolayer MPMs.

### 4.5. Cell Viability

The CCK-8 method was used to evaluate the cytotoxicity of HIDfsin2 on MPMs and THP-1^PMA^ cells. Then, 100 μL of cell suspension containing 6 × 10^4^ cells was used to seed a 96-well plate for 24 h, and then 10 μL volumes of different concentrations of HIDfsin2 (0, 5, 10, and 20 μM) were added to the 96-well plate. After incubating the mixtures for 48 h at 37 °C with 5% CO_2_, the original medium was discarded, and 100 μL of fresh RPMI 1640 medium supplementing 10 μL of CCK-8 solution was added to each well; the plates were incubated for 1 h. The absorbance of each experimental well at 450 nm was measured using a BioTek microplate reader (BioTek, Winooski, VT, USA).

### 4.6. Isothermal Titration Calorimetry (ITC)

The energetics of HIDfsin2 interactions with LPS were analyzed using Microcal PEAQ ITC (MicroCal, Northampton, UK). LPS and HIDfsin2 were dissolved in ddH_2_O and treated with a 0.2 μm Millipore syringe filter before titration. The molecular mass of LPS is about 10 kDa. The typical titration process involved 20 μM HIDfsin2 titrated with 200 μM LPS; the volume of the LPS solution dropped each time was 2 μL, with a total of 19 drops. The sample cell was titrated at 750 rpm until saturated. The value after titration was subtracted from the thermodynamic value of titrating water with LPS. MicroCal PEAQ-ITC analysis software v1.41 was used to calculate the corresponding thermodynamic parameters, such as the binding constant, stoichiometry, N; Gibbs free energy, ∆G; enthalpy change, ∆H; and entropy change, ∆S.

### 4.7. Dynamic Light Scattering (DLS)

The molecular particle size distribution was measured using a Zetasizer Nano ZSP by Malvern (Malvern, UK). PBS solution was processed through a 0.2 μm Millipore syringe filter. LPS micelles were prepared in PBS with pH 7.4 and made to a final concentration of 140 μg/mL. The prepared sample was treated in a 60 °C ultrasonic water bath for 30 min, which was then dropped to room temperature. After 3–4 cycles of heating and cooling, the lipid sample was stored at 4 °C for more than 12 h. After the preparation of aggregates, HIDfsin2 with a final concentration of 28 μM was added to the lipid sample. The two substances were mixed and left at room temperature for 30 min. All of the measurements were completed at 25 °C, and back-scattering detection occurred at 173°. Each value was the average of three independent measurements, and the data for particle size distribution were analyzed using the CONTIN method.

### 4.8. Zeta Potential

The zeta potential was measured using the phase analysis light scattering principle with a Zetasizer Nano ZSP by Malvern (Malvern, UK). LPS was dissolved in ddH_2_O with a final concentration of 50 μM. Similarly, HIDfsin2 was dissolved in ddH_2_O, and the HIDfsin2:LPS molar ratio was controlled in the range of 0–5 and left to act for 1 h. The zeta potential was obtained by substituting the Helmholtz–Smoluchowski formula for the mobility of LPS. The results were the averages of three measurements.

### 4.9. Circular Dichroism (CD) Spectroscopy

The secondary structures of LPS and HIDfsin2 were determined using CD spectroscopy on a Chirascan V100 (AppliedPhotophysics, London, UK). All the samples were dissolved in ddH_2_O and had a final concentration of 50 μM. The spectra were recorded at wavelengths of 180 to 260 nm at room temperature. After subtracting the corresponding blank spectrum, the CD spectrum was collected from the average of the three scans. The results are shown as the mean residue weight (MRW) molar ellipticity [θ] (deg·cm^2^·dmol^−1^).

### 4.10. Cellular Immunofluorescence

The cells were plated on a confocal dish and starved in serum-free medium for 12 h before use. LPS and different concentrations of HIDfsin2 were co-incubated at 37 °C for 2 h, which were then added to the cells. After 30 min, the RPMI 1640 medium was washed away and the cells were washed with PBS 3 times (5 min each time). Next, the cells were fixed with 4% paraformaldehyde tissue fix solution for 10 min at room temperature, and the residual solution was washed away with PBS and treated with 0.1% Triton X-100 permeating solution for 6 min; then, the cells were blocked in PBS solution containing 5% bovine serum albumin (BSA) for 30 min. Subsequently, NF-κB p65 antibody was diluted at 1:200 in a PBS solution containing 1% BSA and incubated overnight at 4 °C. The next day, the cells were incubated with Alexa Fluor 488 secondary antibody (Proteintech, Rosemont, IL, USA, item no. srbAF488-1) for 1 h at 25 °C, and then the cells were stained with DAPI (Thermo Fisher, Waltham, MA, USA, item no. 62248). Cell fluorescence was observed using a confocal laser-scanning microscope (Leica, Solms, Germany, Leica SP8). Alexa 488 has an excitation wavelength of 488 nm, and DAPI has an excitation wavelength of 408 nm.

### 4.11. Quantitative Real-Time PCR (qRT-PCR)

The intracellular total RNA was extracted according to the RNAiso reagent instructions (Takara, Shiga, Japan, item no.9109). Here, 1 μg of total RNA was reverse-transcribed to cDNA using a HiScript II 1st strand cDNA synthesis kit (Vazyme, Nanjing, China) and stored at −20 °C. The upstream and downstream primers (Table 1) were synthesized, and the target DNA was amplified using the SYBR Green method. The experimental results of the qRT-PCR were analyzed using the relative quantitative method (ΔΔCT). The RT-PCR primers are shown in Table 1. All of the experiments were performed no less than three times. The error bars in all the figures present the means ± standard deviations.

### 4.12. Western Blotting

The cells were lysed with 1% sodium dodecyl sulfate (SDS) solution containing protease and phosphatase inhibitors. The total protein content was determined using the BCA protein quantification kit (Vazyme, Nanjing, China, item no. E112-01). Then, 10% SDS-PAGE was prepared, and 20–40 μg of total protein samples was added to each lane. The proteins on the PAGE gel were further transferred to nitrocellulose (NC) membranes, which were then blocked with TTBS solution containing 5% skim milk for 2 h at 25 °C. NC membranes were incubated with 5% skim milk solution containing primary antibody overnight at 4 °C. Subsequently, the primary antibody solution was washed away, and the membranes were reincubated with 3% skim milk solution containing HRP-conjugated secondary antibody for 2 h at room temperature. Finally, the results were visualized and analyzed using Clarity™ Western ECL Substrates (Bio-Rad, Hercules, CA, USA, item no. #1705060) and FuJi medical X-ray film.

### 4.13. Data Analysis

All of the experiments were repeated at least three times. P values were analyzed using two-tailed Student’s unpaired t-tests, and the data were visualized using GraphPad Prism 8 and Adobe Photoshop CS6 v14.0 software. Error bars are shown as means ± SDs. No significance is represented by “ns”, *p* values < 0.05 are marked by stars, *p* values < 0.01 are indicated by two stars, *p* values < 0.001 are indicated by three stars, and *p* values < 0.0001 are indicated by four stars.

## 5. Conclusions

This study suggests that the TLR4 signaling pathway of mouse macrophages responded to the tick saliva peptide HIDfsin2 and further promoted the activation of both NF-κB and type I interferon signaling pathways, thereby inhibiting tick-borne SFTSV replication. These findings are consistent with our previous report that HIDfsin2 hijacked and activated p38 MAPK, promoting tick-borne SFTSV replication in A549 and Huh7 cells with low expression of TLR4. Our study provides new insights into host innate immune processes and has the potential to guide the development of new methods for the emergency diagnosis and prevention of tick-borne diseases.

## Figures and Tables

**Table 1:** Primers for qRT-PCR.

Genes	Forward Primers (5′–3′)	Reverse Primers (5′–3′)
SFTSV	ATGTCAGAGTGGTCCAGGA	TCTCCACCTGTCTCCTTCAG
β-actin	CCGTGAAAAGATGACCCAGA	TACGACCAGAGGCATACAG
TNF-α	TGATCCGCGACGTGGAA	ACCGCCTGGAGTTCTGGAA
IL-1β	ACTCCTTAGTCCTCGGCCA	CCATCAGAGGCAAGGAGGAA
IL-6	GAGGATACCACTCCCAACAGACC	AAGTGCATCATCGTTGTTCATACA

## Data Availability

The original contributions presented in this study are included in the article; further inquiries can be directed to the corresponding authors.
